# Glioblastoma With a Primitive Neuronal Component: A Case Report and Review of the Literature

**DOI:** 10.7759/cureus.108938

**Published:** 2026-05-15

**Authors:** Rui Omichi, Kosuke Nakajo, Yudai Tanoue, Nozomi Tsujino, Kenichi Kohashi, Tsutomu Ichinose, Takeo Goto

**Affiliations:** 1 Neurosurgery, Osaka Metropolitan University Hospital, Osaka, JPN; 2 Neurosurgery, Osaka Metropolitan University, Osaka, JPN; 3 Pathology, Osaka Metropolitan University, Osaka, JPN

**Keywords:** bevacizumab, glioblastoma, idh, primitive neuronal component, stupp protocol

## Abstract

Glioblastoma (GBM) with a primitive neuronal component (PNC) is an uncommon GBM subtype. We report a rare case of GBM with PNC originating in the left frontal lobe and review the relevant literature. A 60-year-old woman with no previous medical history presented with a generalized seizure. MRI revealed a lesion with ring enhancement in the left frontal lobe, suggestive of a high-grade glioma. The patient underwent gross total tumor resection. Intraoperative pathological examination suggested a high-grade glioma; therefore, photodynamic therapy was applied to the resection cavity. Histopathological examination revealed an isocitrate dehydrogenase-wild-type GBM with PNC. The patient received chemoradiation therapy with temozolomide following the Stupp protocol. However, the tumor recurred rapidly within five months after resection, and bevacizumab was initiated. The tumor decreased in size after bevacizumab administration, and no distant metastasis was confirmed on whole-body CT for at least 25 months. GBM with PNC is a newly recognized and rare GBM subtype characterized by early onset, poor prognosis, and a higher propensity for extracranial metastasis and CSF dissemination compared with conventional GBM.

## Introduction

Glioblastoma (GBM) is the most common malignant brain tumor in adults, accounting for nearly 50% of all cases [1]. Due to its rapid infiltrative spread within the CNS, median survival is only 14.6 months despite current standard treatments [2]. GBM with a primitive neuronal component (PNC) is one of the GBM subtypes possessing both classical GBM and embryonal-like histological features [3]. This subtype is very rare, accounting for only 0.5% of all GBM cases [4]. This entity was previously referred to as GBM with primitive neuroectodermal tumor until it was renamed in the 2016 WHO classification of CNS tumors. Also, since the 2021 WHO classification of CNS tumors defined GBM as an isocitrate dehydrogenase (IDH)-wild-type astrocytic tumor [5], some of the previous reports do not reflect this criterion. To the best of our knowledge, no literature review using the latest GBM criteria exists. This work aims to present a rare case of GBM with PNC and review the literature focusing on IDH-wild-type tumors.

## Case presentation

**Figure 1:**
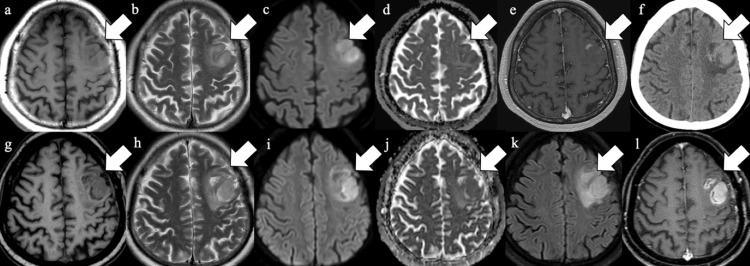
Imaging features The tumor showed slight hypo-intensity on MRI T1-weighted images (white arrow) (a), hyperintensity on T2-weighted images (white arrow) (b), iso- to hyperintensity on diffusion-weighted images (white arrow) (c), and iso- to hyperintensity on apparent diffusion coefficient images (white arrow) (d). Part of the tumor showed homogeneous enhancement after contrast administration (white arrow) (e). CT and MRI evaluated at our hospital (white arrows) (f-l). Brain CT revealed a high-density lesion in the left frontal lobe (white arrow) (f). The tumor showed slight hypointensity on T1-weighted images (white arrow) (g), hyperintensity on T2-weighted images (white arrow) (h), hyperintensity on fluid-attenuated inversion recovery images (white arrow) (i), iso- to hyperintensity on diffusion-weighted images (white arrow) (j), and iso- to hyperintensity on apparent diffusion coefficient images (white arrow) (k). The tumor showed heterogeneous enhancement after contrast administration (white arrow) (l).

The patient was a 60-year-old right-handed woman who presented with a generalized seizure and mild motor aphasia. She had no notable medical history. The tumor showed slight hypo- to isointensity on T1-weighted images (Figure 1a), hyperintensity on T2-weighted images (Figure 1b), hyperintensity on diffusion-weighted images (Figure 1c), and iso- to hyperintensity on apparent diffusion coefficient images (Figure 1d). Part of the tumor showed homogeneous enhancement after contrast administration (Figure 1e). Serum tumor markers, including CEA, CA19-9, SCC, and soluble IL-2 receptor, were negative. Whole-body CT revealed no evidence of extracranial malignancy. The patient underwent a biopsy, but a definitive diagnosis could not be made. She was subsequently followed up for approximately six months, after which tumor enlargement was observed, and she was referred to our hospital.

Brain CT revealed a high-density lesion in the left frontal lobe (Figure 1f). The tumor showed slight hypointensity on T1-weighted images (Figure 1g), hyperintensity on T2-weighted images (Figure 1h), hyperintensity on fluid-attenuated inversion recovery images (Figure 1i), iso- to hyperintensity on diffusion-weighted images (Figure 1j), and iso- to hyperintensity on apparent diffusion coefficient images (Figure 1k). The tumor showed heterogeneous enhancement after contrast administration (Figure 1l).

The patient underwent gross total tumor resection with the aid of a neuronavigation system and motor-evoked potential monitoring. Intraoperative pathological examination suggested a high-grade glioma; therefore, photodynamic therapy was applied around the resection cavity. Postoperative MRI confirmed complete resection with no obvious areas of hyperintensity on diffusion-weighted images.

Postoperatively, the patient developed mild transient right hemiparesis, dysarthria, and motor aphasia but was discharged without neurological deficits two weeks after surgery.

**Figure 2:**
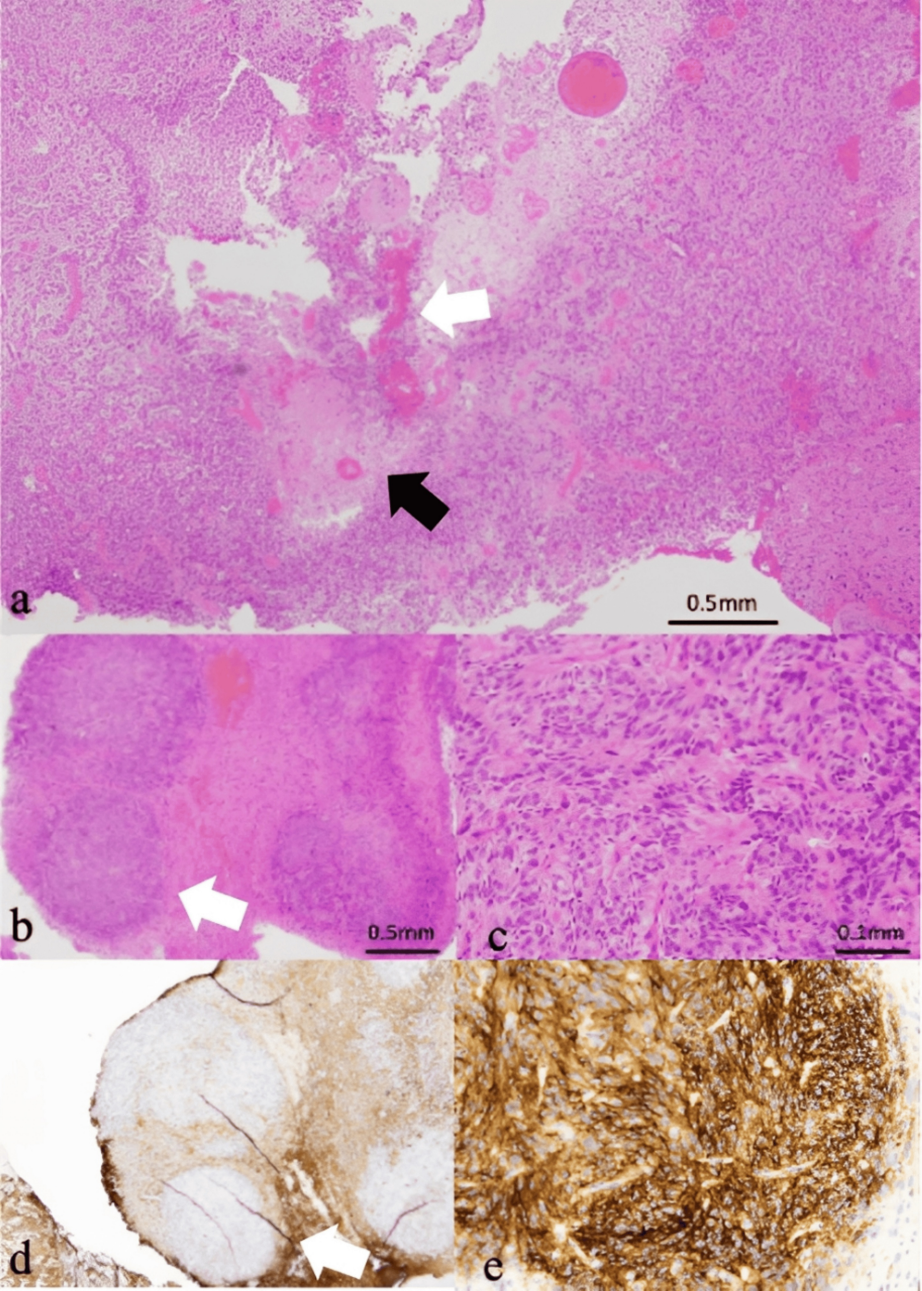
Histopathological features Histopathological examination revealed that the conventional GBM components consisted of diffusely infiltrating hypercellular tumor cells with mitosis, necrosis (black arrow), and prominent microvascular proliferation (white arrow) with pseudopalisading (H&E staining) (a). PNC forms multiple well-demarcated nodules (white arrow) (H&E staining) (b). The PNC resembled embryonal tumors, showing small, round, or spindle-shaped, hypercellular cells with scant cytoplasm and hyperchromatic oval nuclei (H&E staining) (c). The nodule with PNC was negative for GFAP (white arrow) (d) and positive for SSTR-2 (e). GBM, glioblastoma; PNC, primitive neuronal component

Histopathological examination revealed two distinct tumor components forming multiple well-demarcated nodules (Figure 2a). The conventional GBM components consisted of diffusely infiltrating hypercellular tumor cells with mitoses, necrosis with pseudopalisading, and prominent microvascular proliferation. Tumor cells were pleomorphic, including multinucleated giant cells. Another component was composed of primitive neuronal cells resembling embryonal tumors (Figure 2b), consisting of small round or spindle-shaped hypercellular cells with scant cytoplasm and hyperchromatic oval nuclei (Figure 2c). 

Immunohistochemically, the GBM component was positive for GFAP with intact ATRX nuclear expression and negative for IDH1. The PNC cells were negative for GFAP (Figure 2d) and EMA but positive for SSTR-2 (Figure 2e). The Ki-67 labeling index was 9.7%.

Molecular testing revealed negative results for IDH1/2, H3K27M, H3G34R, and MGMT methylation but was positive for TERT promoter mutation. Comprehensive genomic profiling (GenMine TOP) identified mutations in the TERT promoter, FOXA1, HERC2, MSH6, PIK3CA, SPTA1, TOP2A, and FGFR3-TACC3 fusion.

A final diagnosis of IDH-wild-type GBM with PNC was made. The patient received postoperative radiotherapy with concomitant temozolomide, followed by maintenance temozolomide and tumor-treating fields therapy. However, the tumor recurred rapidly within five months. The tumor decreased in size after bevacizumab administration, and no distant metastasis was confirmed on whole-body CT for at least 25 months.

## Discussion

GBM with a PNC is a newly added rare subtype of GBM introduced in the 2016 WHO classification of CNS tumors, accounting for 0.5% of GBM cases [4]. More than 100 cases have been reported since Perry et al. described a series of 53 patients in 2009 [6]. GBM with PNC tends to metastasize to extracranial organs such as the lung and spine and frequently disseminates via the CSF. Patients generally have a worse prognosis, with a median survival of 9.1 months, compared with 14.6 months for conventional GBM [2,6]. However, as the 2021 WHO classification defines GBM as exclusively IDH-wild-type [5], earlier reports consequently included both IDH-mutant and IDH-wild-type tumors. To date, including the present case, 13 cases of IDH-wild-type GBM with PNC have been reported [7-16] (Table 1).

**Table 1:** Literature review of 13 cases of GBM-PNC EOR, extent of resection; GBM, glioblastoma; GTR, gross total resection; MVP, microvascular proliferation; OS, overall survival; PNC, primitive neuronal component; PR, partial resection; RT/TMZ, radiotherapy and temozolomide; STR, subtotal resection

Series	Age/sex	Comorbidity	Side	Location	Intervention timing	EOR	RT/TMZ	Other chemotherapy	TERT promoter status	Necrosis/MVP	MGMT methylation	Distant metastasis	OS
Hendrych et al. (2023) [7]	43/F	Organic psychosyndrome	Left	Frontal lobe	NA	GTR	+/+	-	Wild type	+/+	-	Spine	Eight months
Kumagai et al. (2023) [8]	73/F	Leukemia	Left	Frontal lobe	NA	NA	+/+	-	Wild type	-/-	NA	None	Four months
Sánchez-Ortega et al. (2020) [9]	77/F	Hypertension, dyslipidemia, atrial fibrillation	Right	Frontoparietal lobe	NA	GTR	+/+	-	NA	NA/NA	+	None	Two weeks
Ma et al. (2023) [10]	57/F	Diabetes mellitus	Left	Parieto-occipital temporal lobe	NA	NA	+/+	-	NA	+/NA	NA	None	NA
Poyuran et al. (2021) [11]	11/F	NA	Right	Frontoparietal lobe	NA	STR	NA	-	NA	+/+	NA	None	NA
	48/M	NA	Right	Frontoparietal lobe	NA	GTR	+/+	-	NA	+/+	NA	None	More than 10 months
Donabedian et al. (2021) [12]	62/F	NA	Left	Thalamus	NA	PR	+/-	-	NA	+/+	-	None	Six months
	52/F	-	Left	Frontal lobe	NA	GTR	+/-	Pembrolizumab	NA	+/+	+	None	NA
Tan et al. (2017) [13]	Three months/F	-	Right	Frontotemporal lobe	NA	STR	-/-	Carboplatin, etoposide, cyclophosphamide	NA	+/+	NA	None	More than seven months
Rong et al. (2021) [14]	20/M	NA	Left	Temporal lobe	Four weeks later	GTR	+/+	-	Wild type	NA/NA	-	Spine, pelvic, and femur	15 months
Vollmer et al. (2019) [15]	47/M	Acute leukemia	Right	Temporal lobe	NA	STR	+/+	-	NA	+/+	NA	Spine	NA
Tamai et al. (2019) [16]	49/M	NA	Right	Temporal lobe	NA	GTR	+/+	Bevacizumab	Mutant	+/+	+	Spine and lung	12 months
Present case	60/F	-	Left	Frontal lobe	Soon	GTR	+/+	Bevacizumab	Mutant	+/+	-	None	More than 25 months

Our literature review revealed that this tumor occurs at a younger age. Even excluding two pediatric patients, the average age of patients was 53.5 (95% CI: 44.7-62.2; range: 20-77 years), which is younger than that of conventional GBM (median: 64 years) [17]. A possible explanation is the presence of primitive neuronal elements or embryonal tumor components, which typically occur in childhood and may lower the age of onset. There was a female predominance (female: male = 9:4). Two cases had a history of leukemia; however, intervention timing was not clearly described in most cases. Clinical features, tumor location, and imaging findings were similar to those of conventional GBM. All tumors originated in the frontal and/or temporal lobes. The present case involved a 60-year-old female, who was still younger than the typical conventional GBM cases. The other factors mentioned above did not allow a clear distinction from conventional GBM, as in other reported cases.

Ten out of 13 cases showed the essential histological features of GBM, including necrosis and microvascular proliferation, although genetic mutations such as TERT promoter status and MGMT methylation were assessed only in some cases. GBM with PNC contains both a typical GBM component and an embryonal-like component; however, the tumor origin remains unclear, with proposed hypotheses including (1) development of a differentiated glial tumor from pre-existing neuronal cells [13]; (2) neuronal metaplasia or dedifferentiation of the astrocytic component resulting in neuronal cells [13]; (3) collision of two distinct clonal expansions [18]; and (4) development of both components from a common stem cell population [19].

Overall survival is worse than that of conventional GBM. Nine cases reported survival time, with survival less than 15 months in all cases except the present case. A possible reason for the poor prognosis is resistance to temozolomide, which is often ineffective against PNC [6]. Therefore, Perry et al. recommended platinum-based chemotherapy after temozolomide failure [6]. In addition, the absence of MGMT methylation generally limits temozolomide efficacy. In fact, only 3 (23.1%) cases showed MGMT methylation. In the present case, MGMT methylation was negative, and the patient showed early recurrence despite standard temozolomide therapy. We administered bevacizumab, and FGFR3-TACC3 fusion-targeted therapy was considered, although platinum-based chemotherapy might have been preferable. In our literature review, one patient who received platinum chemotherapy showed no neurological deficits or recurrence for at least seven months [13].

Metastatic potential is another distinctive feature of IDH-wild-type GBM with PNC. Of the 13 reviewed cases, four (30.8%) showed metastasis to the lung or spine, which is relatively high compared with the reported incidence of 0.4-2% in conventional GBM [20,21]. Other studies have reported systemic GBM metastases in 6-27% of autopsy series [22,23], but the accuracy of these estimates is uncertain because the short survival time may be insufficient for metastases to become clinically apparent. However, the reported metastasis rate in IDH-wild-type GBM with PNC is as high as 30%. Hendrych et al. reported cervical spine metastasis in a 43-year-old woman five months after tumor resection and suggested that NF1, NOTCH3, and ARID1A mutations may contribute to acquired invasiveness and metastatic potential [7]. Rong et al. reported a 20-year-old man who presented with a sudden onset of intratumoral hemorrhage in the left temporal lobe, with cervical, thoracolumbar spine, pelvic, and femoral metastases [14]. Vollmer et al. described an unusual case of GBM with PNC in a 47-year-old man with a past history of brain irradiation for acute leukemia during childhood who later developed metastasis to the cervical spine [15]. Only one report described a case presenting with both lung and multiple spinal metastases in a 49-year-old man [16]. Overall, these findings suggest that routine whole-body imaging may be warranted during follow-up. Accordingly, we regularly perform whole-body CT as well as brain imaging. Fortunately, the patient has shown no recurrence or metastatic lesions and has survived for more than 25 months since the initial treatment. This study has some limitations. The primary limitation is the small sample size. Second, as many publications lack genetic information, such as TERT promoter status and MGMT methylation, this limits genetic-level analysis.

## Conclusions

We report a rare case of GBM with PNC. The classification of this entity has evolved over time following its renaming in 2016 and the updated GBM definition in 2021. We reassess this disease as being characterized by earlier onset, poor prognosis, and a higher propensity for extracranial metastasis and CSF dissemination compared with conventional GBM. Therefore, careful clinical management is required, and whole-body imaging surveillance is recommended. As our study is limited by a small sample size and incomplete genetic information in the existing literature, further case accumulation is needed to clarify its pathogenesis and establish optimal treatment strategies.

## References

[1] Ostrom QT, Cioffi G, Waite K, Kruchko C, Barnholtz-Sloan JS (2021). CBTRUS statistical report: primary brain and other central nervous system tumors diagnosed in the United States in 2014-2018. Neuro Oncol.

[2] Stupp R, Mason WP, van den Bent MJ (2005). Radiotherapy plus concomitant and adjuvant temozolomide for glioblastoma. N Engl J Med.

[3] Louis DN, Perry A, Reifenberger G (2016). The 2016 World Health Organization classification of tumors of the central nervous system: a summary. Acta Neuropathol.

[4] Prelaj A, Rebuzzi SE, Caffarena G (2018). Therapeutic approach in glioblastoma multiforme with primitive neuroectodermal tumor components: case report and review of the literature. Oncol Lett.

[5] Louis DN, Perry A, Wesseling P (2021). The 2021 WHO classification of tumors of the central nervous system: a summary. Neuro Oncol.

[6] Perry A, Miller CR, Gujrati M (2009). Malignant gliomas with primitive neuroectodermal tumor-like components: a clinicopathologic and genetic study of 53 cases. Brain Pathol.

[7] Hendrych M, Solar P, Hermanova M (2023). Spinal metastasis in a patient with supratentorial glioblastoma with primitive neuronal component: a case report with clinical and molecular evaluation. Diagnostics (Basel).

[8] Kumagai M, Takata S, Watanabe T (2023). Glioblastoma/high-grade glioma with a primitive neuronal component including rhabdoid differentiation that was difficult to diagnose: a case report. Pathol Res Pract.

[9] Sánchez-Ortega JF, Aguas-Valiente J, Sota-Ochoa P, Calatayud-Pérez J (2020). Glioblastoma with primitive neuronal component: a case report and considerations of fluorescence-guided surgery. Surg Neurol Int.

[10] Ma Q, Liu L, Sun N (2023). Glioblastoma with a primitive neuronal component: a case report. Oncol Lett.

[11] Poyuran R, Chandrasekharan K, Easwer HV, Narasimhaiah D (2021). Glioblastoma with primitive neuronal component: an immunohistochemical study and review of literature. J Clin Neurosci.

[12] Donabedian P, Tuna I, Rahman M, Gregory J, Kresak J, Rees JH (2021). Glioblastoma with a primitive neuroectodermal component: two cases with implications for glioblastoma cell-of-origin. Clin Imaging.

[13] Tan CH, Phung TB, Xenos C (2017). Glioblastoma with primitive neuronal pattern in a girl aged 3 months: a rare diagnosis at an unusual age. BMJ Case Rep.

[14] Rong T, Zou W, Qiu X (2021). A rare manifestation of a presumed non-osteophilic brain neoplasm: extensive axial skeletal metastases from glioblastoma with primitive neuronal components. Front Oncol.

[15] Vollmer K, Pantazis G, Añon J, Roelcke U, Schwyzer L (2019). Spinal metastases of supratentorial glioblastoma with primitive neuronal component. World Neurosurg X.

[16] Tamai S, Kinoshita M, Sabit H (2019). Case of metastatic glioblastoma with primitive neuronal component to the lung. Neuropathology.

[17] Thakkar JP, Dolecek TA, Horbinski C, Ostrom QT, Lightner DD, Barnholtz-Sloan JS, Villano JL (2014). Epidemiologic and molecular prognostic review of glioblastoma. Cancer Epidemiol Biomarkers Prev.

[18] Forbes V, Vredenburgh J (2016). Primitive neuroectodermal tumor with glioblastoma multiforme components in an adult: a collision tumor. Cureus.

[19] Kandemir NO, Bahadir B, Gül S, Karadayi N, Ozdamar SO (2009). Glioblastoma with primitive neuroectodermal tumor-like features: case report. Turk Neurosurg.

[20] Amitendu S, Mak SK, Ling JM, Ng WH (2012). A single institution experience of the incidence of extracranial metastasis in glioma. J Clin Neurosci.

[21] Vertosick FT Jr, Selker RG (1990). Brain stem and spinal metastases of supratentorial glioblastoma multiforme: a clinical series. Neurosurgery.

[22] Onda K, Tanaka R, Takahashi H, Takeda N, Ikuta F (1989). Cerebral glioblastoma with cerebrospinal fluid dissemination: a clinicopathological study of 14 cases examined by complete autopsy. Neurosurgery.

[23] Rosen J, Blau T, Grau SJ, Barbe MT, Fink GR, Galldiks N (2018). Extracranial metastases of a cerebral glioblastoma: a case report and review of the literature. Case Rep Oncol.

